# Leukemic Phase of CD5+ Diffuse Large B-Cell Lymphoma

**DOI:** 10.4274/tjh.2016.0447

**Published:** 2018-08-05

**Authors:** Hui-Hua Hsiao, Hui-Ching Wang, Yu-Fen Tsai, Chien Hsiao, Shih-Feng Cho, Yi-Chang Liu

**Affiliations:** 1 Kaohsiung Medical University Hospital, Internal Medicine; Kaohsiung Medical University, Faculty of Medicine, Kaohsiung, Taiwan

**Keywords:** Lymphoma, Acute leukemia, Flow cytometry

Acute lymphoid leukemia and diffuse large B-cell lymphoma, though categorized as lymphoid neoplasms, have different clinical presentations, treatment protocols, and outcomes. However, the rare situation of a leukemic phase of CD5+ diffuse large B-cell lymphoma sometimes mimics acute lymphoid leukemia and requires careful differentiation. We report here a rapid and accurate diagnosis by flow cytometry. 

A 55-year-old woman suffered from hemoptysis and thrombocytopenia with lymphadenopathies. Complete blood count revealed a white cell count of 10.2x10^9^/L with 46% blast cells. Peripheral blood smear showed marked blastocytosis with fine nuclear chromatin and prominent nucleoli and scanty cytoplasm (Figure 1, left). Flow cytometry showed positive results for CD5, CD19, CD20, and kappa light chain but was negative for CD7, CD10, CD11b, CD13, CD33, CD34, CD56, and terminal deoxynucleotidyl transferase (TdT). Bone marrow examination revealed scattered involvement of CD20-positive and TdT-negative cells (Figures 1 and 2). Biopsy of the neck lymph nodes confirmed the diagnosis of CD5+ diffuse large B-cell lymphoma (Figure 2, lower right). Under the diagnosis of stage IV disease, she received 8 courses of R-CHOP therapy with stem cell transplantation later on. She has sustained complete response after therapy for 2 years to date.

A leukemia phase of diffuse large B-cell lymphoma is rare and mimics acute lymphoblastic leukemia [1,2]. Flow cytometry with an appropriate panel could help in differentiating lymphoma from leukemia [2,3]. In this case, having the surface light chain and TdT markers made for an accurate and rapid diagnosis.

## Figures and Tables

**Figure 1:**
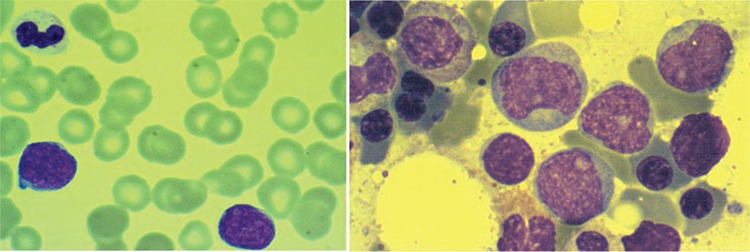
Peripheral blood smear showed thrombocytopenia with marked lymphoid blast-like cells of fine nuclear chromatin with prominent nucleoli and scanty cytoplasm (left: hematoxylin and eosin stain, 1000^x^). Bone marrow examination revealed scattered involvement of median to large cells with prominent nucleoli (right: hematoxylin and eosin stain, 1000^x^).

**Figure 2:**
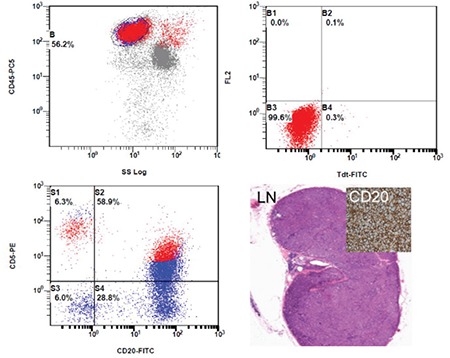
Flow cytometry revealed positivity for CD5 and CD20 with negativity for terminal deoxynucleotidyl transferase (upper and lower left). Lymph node biopsy showed diffuse lymphoma pattern with positivity for CD20 (lower right). TdT: Terminal deoxynucleotidyl transferase.
